# The *Dictyostelium discoideum* FimA protein, unlike yeast and plant fimbrins, is regulated by calcium similar to mammalian plastins

**DOI:** 10.1038/s41598-023-42682-1

**Published:** 2023-09-27

**Authors:** Hiroaki Ishida, Andrew G. Woodman, Naoya Kitada, Tomoyasu Aizawa, Hans J. Vogel

**Affiliations:** 1 Biochemistry Research Group, Department of Biological Sciences, University of Calgary, Calgary, AB T2N 1N4 Canada; 2 Faculty of Advanced Life Science, Hokkaido University, Sapporo, 060-0810 Japan

**Keywords:** Proteins, Structural biology

## Abstract

Plastins, also known as fimbrins, are highly conserved eukaryotic multidomain proteins that are involved in actin-bundling. They all contain four independently folded Calponin Homology-domains and an N-terminal headpiece that is comprised of two calcium-binding EF-hand motifs. Since calcium-binding has been shown to be integral to regulating the activity of the three mammalian plastin proteins, we decided to study the properties of the headpiece regions of fimbrins from the model plant *Arabidopsis thaliana*, the yeasts *Saccharomyces cerevisiae* and *Schizosaccharomyces pombe* and the amoeba *Dictyostelium discoideum*. Of these protein domains only the FimA headpiece from the amoeba protein possesses calcium binding properties. Structural characterization of this protein domain by multidimensional NMR and site-directed mutagenesis studies indicates that this EF-hand region of FimA also contains a regulatory ‘switch helix’ that is essential to regulating the activity of the human L-plastin protein. Interestingly this regulatory helical region seems to be lacking in the plant and yeast proteins and in fimbrins from all other nonmotile systems. Typical calmodulin antagonists can displace the switch-helix from the FimA headpiece, suggesting that such drugs can deregulate the Ca^2+^-regulation of the actin-bunding in the amoeba, thereby making it a useful organism for drug screening against mammalian plastins.

## Introduction

Plastins and fimbrins are a class of proteins that are found in all eukaryotes. They have uncommon, yet conserved, domain structures, containing four independently folded Calponin Homology (CH)-domains that are preceded by two Ca^2+^-binding EF-hand motifs^1^. It is well known that two adjacent CH-domains together make up an actin-binding motif. Hence, plastins and fimbrins contain two adjoining actin binding motifs, allowing them to bind two actin filaments simultaneously, leading to overall actin bundling^2^. The three human plastin proteins are expressed differentially in distinct tissues. In healthy individuals the L-plastin protein is normally only expressed in hematopoietic cells; it is also known as lymphocyte cytosolic protein (LCP-1)^3,4^. The T-plastin protein is expressed in all solid tissues, while the more recently discovered I-plastin protein is expressed selectively in the intestine and the kidney^5^. I-plastin is also found abundantly in vestibular hair cell stereocilia^6^. These three related proteins are expressed from genes that are located on different chromosomes, chromosome 3 (I-plastin), chromosome 13 (L-plastin) and chromosome X (T-plastin), respectively. Based on their extensive amino acid sequence homology and the similar organization of these three genes in introns and exons, it seems likely that the three proteins have arisen from a common ancestor. Interestingly, these three differentially expressed plastins are found amongst all vertebrates^1^. However, the plastins, which are also known as fimbrins, are conserved from lower eukaryotes to humans. Unique fimbrins have been found and characterized in the yeasts *Saccharomyces* cerevisiae and *Schizosaccharomyces* pombe, that are called Sac6P^7–10^ and Fim1^11,12^, respectively. Similar proteins have been found in *Aspergillus nidulans*^13^ and in pathogenic fungi such as *Candida albicans*^14^ and *Cryptococcus neoformans*^15^. Moreover, fimbrins have also been described in various plants, and the roles of the AtFim1, AtFim4 and AtFim5 proteins from the model plant *Arabidopsis thaliana*, have already been studied in some detail^16–20^.

The plastin and fimbrin proteins are multidomain proteins that are build-up of distinct independently folded units. They are comprised of two N-terminal calcium-binding EF-hand motifs that are followed by four CH-domains. This unique domain architecture, with four CH-domains, has not been found in any other proteins. CH-domains are independently folded highly helical structures, of about 120 amino acids, that are found approximately 90 times in the human genome. It is well-known that two tandem CH-domains together make up an actin binding motif as is seen in many other multidomain actin-binding proteins, such as actinin, spectrin and dystrophin for example^21^. Because all the plastins and fimbrins have four CH-domains they all contain two Actin Binding Domains (ABD) and hence they can bridge between two F-actin strands, thereby giving rise to actin bundling. It seems likely that all the plastins and fimbrins from lower eukaryotes to humans must play a role in actin bundling, as the four CH-domain architecture is strictly conserved across all species. Indeed, the crystal structure of the four CH-domains of the yeast and plant proteins shows that they are arranged in a horsehoe-like structure^2^, where the CH1 and CH4 domains interact with one another. The same compact structure for the four plastin CH-domains is maintained in aqueous solution as well, as demonstrated by small angle X-ray scattering studies^22^. Previous Cryo-EM studies have shown that this domain orientation could allow the protein to bind two actin strands in a parallel manner, giving rise to actin-bundling^23–27^. Furthermore, recent studies have proposed regulatory mechanisms for the actin-bundling via calcium-binding and/or protein phosphorylation^28,29^.

When the similarities between the chicken intestinal fimbrins and the human leukocyte plastins were originally recognized^30^, the two proteins were described as having domains that are homologous with calmodulin and actin-binding proteins. Calmodulin is a well-known calcium-regulatory protein that can bind four calcium ions and regulate the activity of many target proteins^31^. As a result, it was not totally unexpected when it was shown that L-plastin could bundle actin in a calcium-dependent manner, where increased calcium levels led to inhibition of the actin-bundling activity^32^. Although the two EF-hand motifs, that together would make up one half of calmodulin, are preserved in plastins from all species, several mutations can be found in the calcium binding loops of all plant and yeast proteins that would likely abolish effective calcium binding and hence it is predicted that these are not capable of binding calcium^33^. The solution structure of the EF-hand containing headpiece region of human L-plastin was recently determined by NMR spectroscopy^34^. The protein domain was folded like a single lobe of calmodulin, and similar to most regulatory EF-hand calcium-binding proteins^35^. In the apo-state the four helices that are contributed by the two helix-loop-helix EF-hands, are oriented in a parallel manner, and the structure undergoes a typical helix reorientation and overall opening following the binding of calcium. Intriguingly the target for this calcium-binding domain was not located in one of the four CH-domains, as had originally been expected, but it was located in the unstructured region between the two EF-hand domains and the first CH-domain. This region folds into a well-defined α-helix when it binds to the holo-form of the headpiece. Consequently, this regulatory region has been termed the switch helix, as it appears to be involved in switching the actin-bundling on-and-off. Recent studies have shown that the same mechanism, with a regulatory switch helix, also occurs in the human T-plastin headpiece, albeit at slightly different calcium concentrations^36–38^.

In this work we have studied the headpiece regions of plastins and fimbrins obtained from different species, such as yeasts, plants and the amoeba *Dictyostelium* discodeum^33,39^. The latter protein was included in our work because earlier reports had suggested that the amoeba FimA protein, like the animal plastins, was also regulated in a calcium-dependent manner^40^. Using NMR and CD spectroscopy, we could show that none of the four plant and yeast headpieces were responding to calcium, even though all constructs were well folded. In contrast, the headpiece region of the amoeba protein responded to calcium in a manner that resembled the results obtained with the human L-plastin protein. We subsequently analyzed the structure of the FimA headpiece in more detail and studied the interaction with its own switch helix. Taken together our results show that the amoeba FimA protein can be regulated in a similar calcium-dependent manner as the human plastins, making this organism a potentially suitable target for screening drug fragments and potential drugs directed towards inhibiting L-plastin-mediated cancer metastasis.

## Results

### Nuclear magnetic resonance (NMR) studies of fimbrin headpiece domains

**Figure 1:**
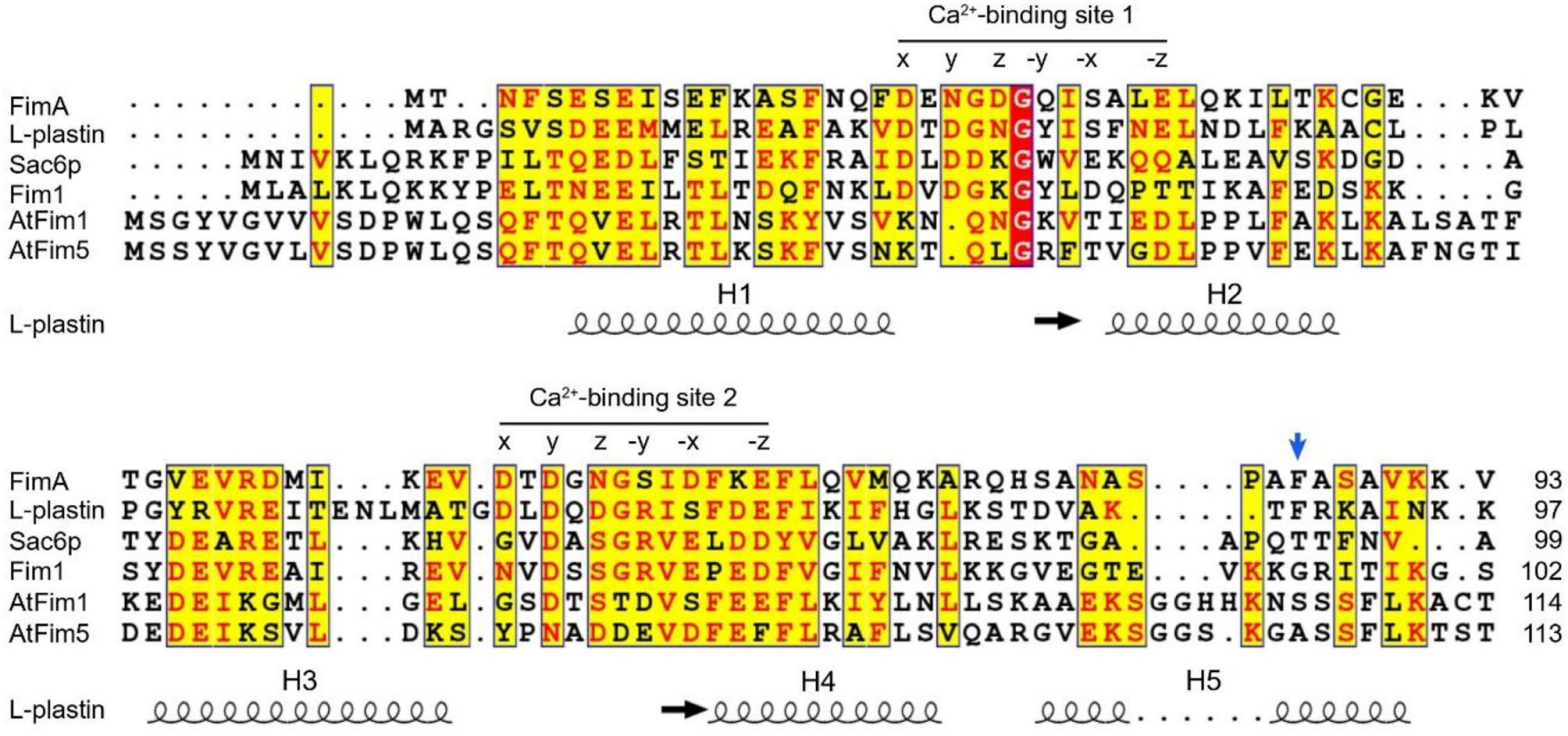
Sequence alignment of the headpiece domains of various fimbrins and human L-plastin. Identical residues are boxed in red. Similar residues are boxed in yellow and highlighted in red. A schematic drawing of the experimentally determined secondary structure elements of L-plastin is also included. The regions with coils and arrows indicate α-helices and β-sheets, respectively. The helices are labeled as in the text. The position of the key hydrophobic residue (F86) is indicated by an arrow. UniProtKB accession numbers are FimA, P54680; human L-plastin, P13796; Sac6p, C8Z545; Fim1, O59945; AtFim1, Q7G188; and AtFim5, Q9FKI0. The residues that serve as the calcium ligands in the EF-hand Ca^2+^-binding sites 1 and 2 are indicated by their coordination.

**Figure 2:**
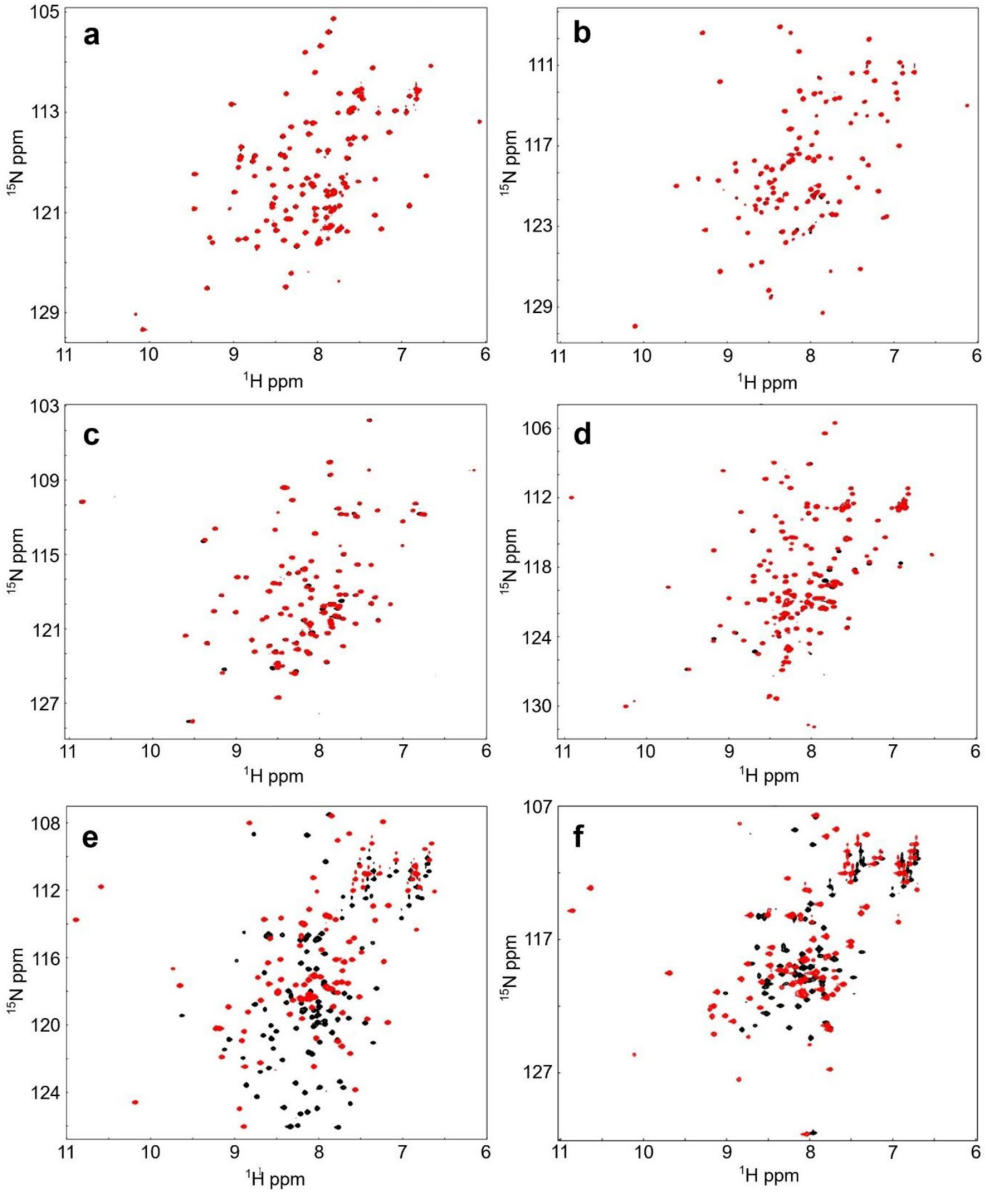
Overlaid ^1^H, ^15^N-HSQC spectra of AtFim1 (**a**), AtFim5 (**b**), Fim1 (**c**), Sac6p (**d**), FimA (**e**), and FimAN81Ter (**f**) in the presence (red) and absence (black) of Ca^2+^.

Our sequence alignment shows that among the fimbrin headpiece regions of five different species including *Saccharomyces cerevisiae* (Sac6p), *Schizosaccharomyces* pombe (Fim1), *Arabidopsis thaliana* (AtFim1 and 5), and *Dictyostelium discodeum* (FimA), only FimA contains functional EF-hand calcium binding sites (Fig. 1)^35^. For example, the positions X, Y, Z provide side-chain oxygen atoms as ligands for the calcium, which typically come from aspartate residues. The glutamate residue found at the –Z position is also an important Ca^2+^ ligand. To confirm this notion, we have expressed and purified all five headpiece domains. All constructs are soluble and can be expressed in the cytoplasm of *E. coli*. ^1^H,^15^N HSQC NMR shows that all spectra were well dispersed, indicating the presence of a well-folded protein domain (Fig. 2). As expected from the amino acid sequences, upon addition of a saturating amount of calcium, no substantial chemical shift changes were observed in the HSQC spectra of AtFim1, AtFim5, Sac6p, and Fim1. Our results confirm that these headpiece domains form an independently folded helical domain but that they are not able to bind calcium ions with micromolar affinities and that they do not undergo the typical conformational change seen in most EF-hand proteins.

On the other hand, drastic structural changes, resembling those reported earlier for human L-plastin (LPL) and T-plastin (TPL) headpiece domains^34,37^, were observed with the headpiece region of FimA (Fig. 2e). Furthermore, our sequence analysis and secondary structure predictions indicate that the FimA handpiece domain, but not other fimbrin domains studied here, is followed by an extra alpha-helix (H5 in Fig. 1), which could correspond to the “switch-helix” region of human LPL that regulates actin-binding in Ca^2+^-dependent manner^34^. Considering the helical regulatory region and the two functional calcium-binding motifs, the FimA headpiece resembles the headpiece domain of human L-plastin (LPL-EF). This observation is in line with the similarities between FimA and LPL reported earlier^40^.

### Structural characterization of the headpiece domain of FimA

**Figure 3:**
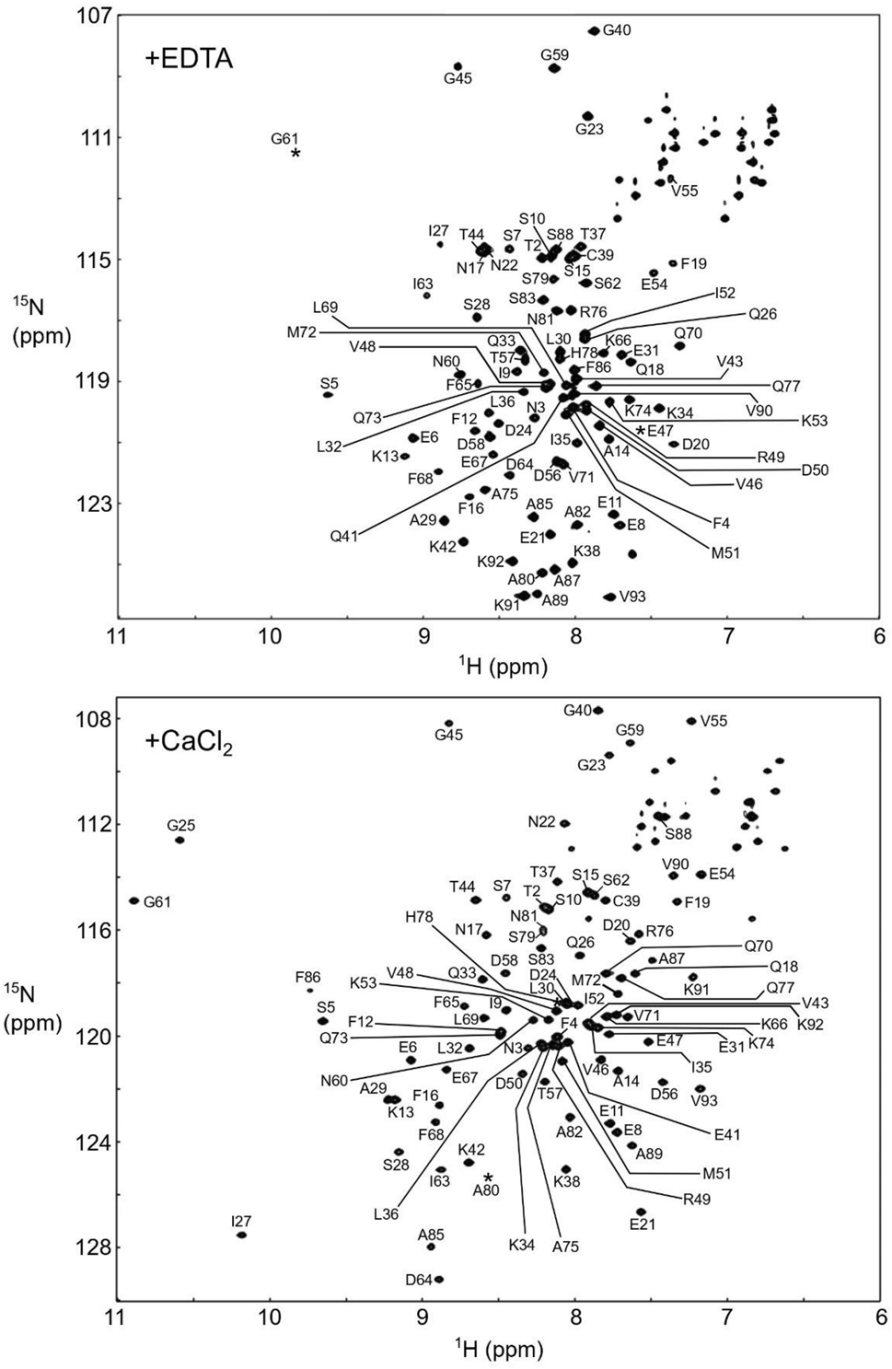
Assigned ^1^H, ^15^N-HSQC spectra of FimA in the presence of EDTA (top) and CaCl_2_ (bottom). The positions of a few signals that are invisible at this counter level are indicated by asterisks.

**Figure 4:**
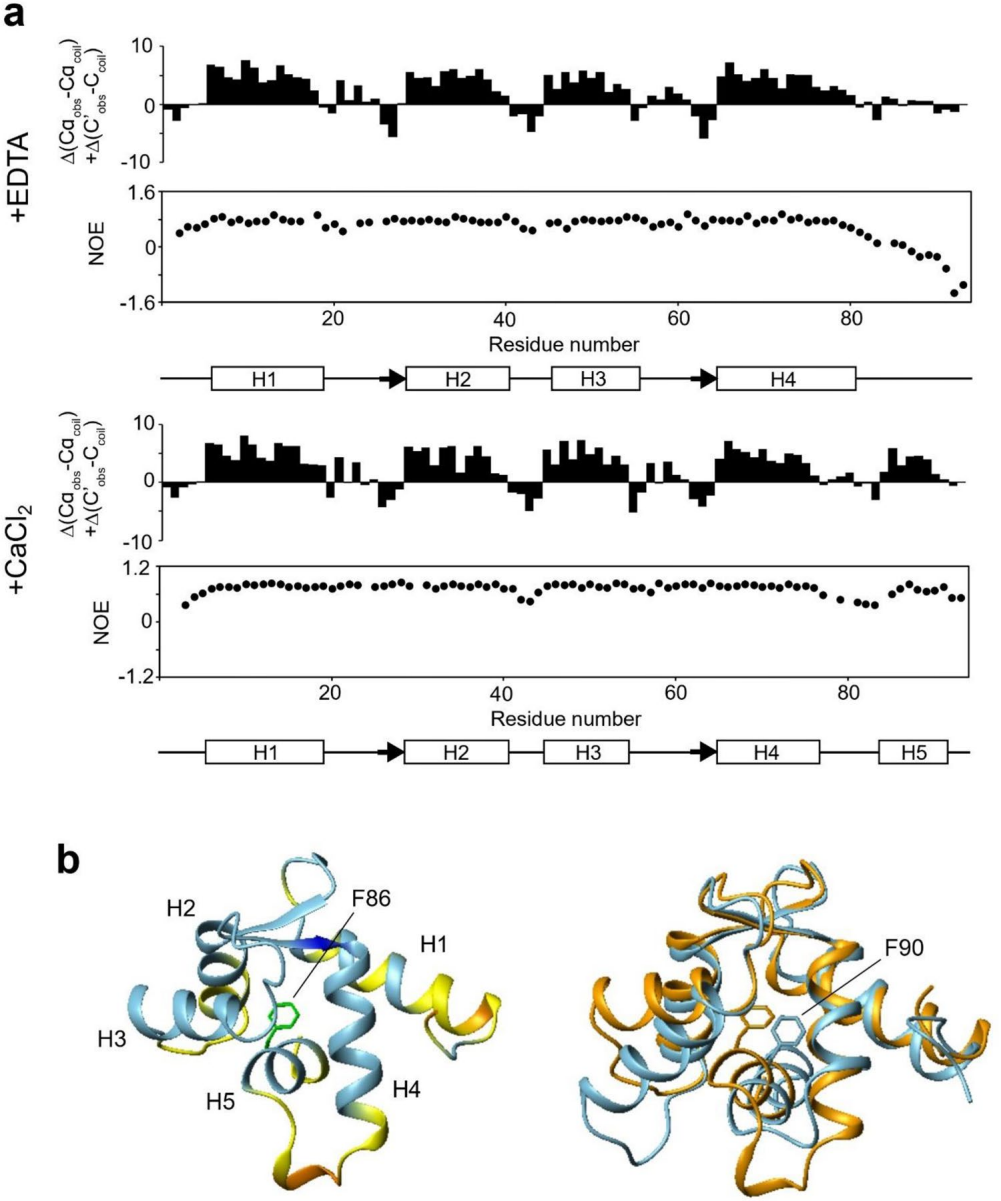
(**a**) Chemical shift index (CSI) and {^1^H}-^15^N heteronuclear NOE values are plotted as a function of the residue number in the presence of EDTA and CaCl_2_. The secondary structures predicted from the CSI are also displayed. (**b**) The predicted structure of FimA-EF was obtained from the AlphaFold database (left). The regions are colored according to a per-residue confidence score of very high (blue), confident (sky blue), low (yellow), and very low (orange). The sidechain of F86 is show. α-helices are labeled. The structures of FimA-EF (orange) and the headpiece domain of human L-plastin (sky blue; 5JOJ) are superposed (right). The Phe residues that serve as an anchor residue are displayed in both structures.

After this initial characterization, we sought to further investigate the calcium regulatory mechanisms of the headpiece domain of FimA (here in after, FimA-EF). For NMR spectroscopy, we successfully expressed and purified the ^13^C, ^15^N-labeled FimA-EF (residues 1–93) and we could unambiguously assign all the backbone amide signals other than one proline residue in the HSQC spectrum of Ca^2+^-bound form (Fig. 3). In the apo-form, the signal from G25 was also not identified likely due to signal broadening. Note that considerably less signals are observed around 8 ppm in the spectrum of the Ca^2+^-bound form (Fig. 2e), indicating that the protein gains a more defined structure when Ca^2+^ is bound. By analyzing the chemical shift index (CSI) values along the protein backbone, we could determine the secondary structures of both the apo- and Ca^2+^-bound forms (Fig. 4a). FimA-EF has four α-helices (H1-H4) consisting of two helix-loop-helix EF-hand Ca^2+^-binding motifs in both forms. {^1^H}-^15^N heteronuclear NOE data shows that the unstructured C-terminal region is flexible in the apo-form (Fig. 4a). However, upon binding Ca^2+^, this region forms another stable α-helix (H5). This could also be observed by CD spectroscopy as an increased helical content (Supplementary Fig. S1), which was also reported for LPL-EF^37^. We then obtained the predicted model structure for FimA-EF from the AlphaFold database (Fig. 4b). The AlphaFold structure of FimA-EF is totally consistent with the secondary structures determined with our NMR data for the Ca^2+^-form. However, it is not in agreement with the apo-form since the H5 is present and interacts with the hydrophobic pocket of the main structure. It seems that the long H4 present in the apo-form needs to be partially unfolded to achieve this interaction (Fig. 4a), which is an effect that was not observed with LPL-EF^34^. Figure 4b also shows the superposition of the structures of FimA-EF and Ca^2+^-bound LPL-EF. Although the global sequence identity between FimA and human LPL is 48%, it is reduced to 27% when only the headpiece domains are compared. However, the positions of all the secondary structure elements are well conserved in the tertiary structures and the backbone RMSD for the regions of FimA-EF (4–57, 62–82, and 83–97) and L-plastin (2–55, 56–76, and 79–93) is only 4.32 Å. The most prominent differences between the two structures would be the orientation of H3 as well as the length of H5. The regulatory H5 helix of FimA (residues 84–90) is much shorter than that of LPL (residues 85–94). This is most likely due to the presence of a Pro residue at position 84 in FimA (Fig. 1). The key hydrophobic residue for the interaction between H5 and the main structure of FimA-EF appears to be the F86 residue that is deeply inserted into the hydrophobic pocket formed by the EF-hand motifs (Fig. 4b), which would correspond to the key hydrophobic F90 residue in human LPL (Fig. 1; Ishida et al.^34^). After this finding, we decided to generate the FimA-EF-F86A mutant for further investigations.

To further characterize the Ca^2+^-induced conformational changes of the EF-hand domain of FimA as well as its interaction with the H5 region, we also generated a truncated construct, FimA-EF-N81ter (residues 1–80) that omits the entire H5 region. Similar to FimA-EF, large spectral changes were observed in the HSQC spectrum upon Ca^2+^-binding (Fig. 2f). We performed the NMR chemical shift assignment for this construct in both the apo- and Ca^2+^-bound forms. Although the assignment of the Ca^2+^-bound form was completed without any ambiguity, that of the apo-form could not be completed due to several missing or very weak signals (Supplementary Fig. S2). By analyzing the CSI values, we could confirm that the secondary structures of Ca^2+^-bound FimA-EF-N81ter retains the same conformation as the FimA-EF despite the absence of H5 (Supplementary Fig. S3).

### Ca^2+^-induced conformational changes monitored by fluorescence spectroscopy

**Figure 5:**
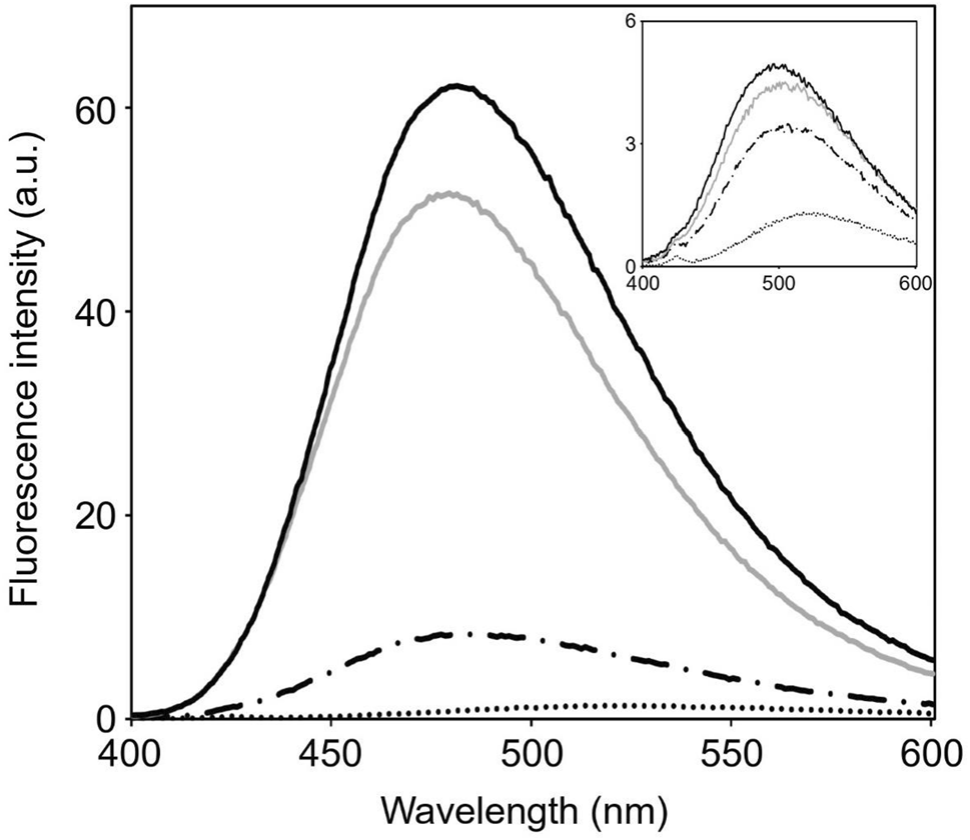
Ca^2+^-induced exposure of the hydrophobic surfaces of EF-hand domain monitored by ANS fluorescence. The steady-state fluorescence emission spectra of ANS in the absence (dotted line) and the presence of FimA-EF (dash-dotted line), FimA-EF-N81Ter (black solid line), FimA-EF-F86A (gray solid line). The spectra in the presence of EDTA are shown in the inset. All experiments were carried out at 25 °C.

To further evaluate the Ca^2+^-induced conformational changes of the EF-hand domain, we performed ANS dye fluorescence experiments with FimA-EF, FimA-EF-N81ter, and FimA-EF-F86A. The fluorescence intensity of ANS is enhanced when it moves from aqueous solution to a non-polar environment, and therefore it can be utilized as a probe to detect hydrophobic surfaces of proteins^41,42^. In the absence of calcium ions, very little enhancement was observed for all three protein domains (Fig. 5). However, in the presence of calcium, the fluorescence intensity with FimA-EF-N81ter was drastically enhanced approx. 60 times, whereas the intensity of FimA-EF remains much like that seen in the presence of EDTA.

From these results, it is reasonable to conclude that the EF-hand domain of FimA transforms from a closed state into an open-conformation in which the previously hidden hydrophobic pocket becomes exposed to the solvent, allowing it in turn to interact with the H5 region of the protein. This sequence of events is analogous to LPL^34^. No fluorescence enhancement is observed with FimA-EF since the H5 region already occupies the hydrophobic pocket and thereby blocks access for ANS. On the other hand, the FimA-EF-F86A mutant showed a fluorescence enhancement that is only slightly smaller than that of FimA-EF-N81ter regardless of the presence of H5. This result confirms that F86 on the H5 helix plays a major role in anchoring the H5 to the hydrophobic pocket of the FimA EF-hand domain. Clearly, by removing the phenyl ring of F86, we could totally disrupt this interaction.

### Interaction between EF-hand domain and H5

**Figure 6:**
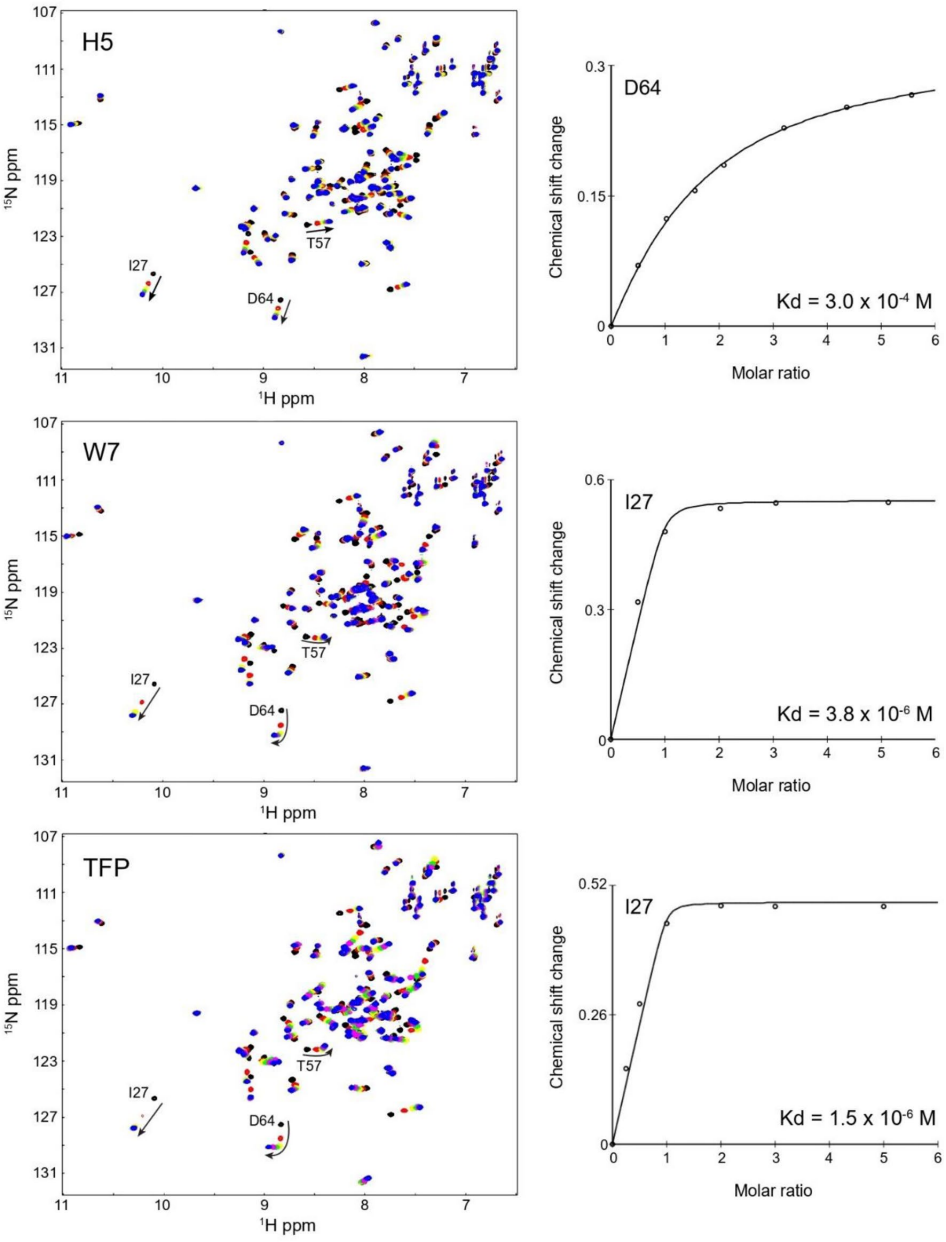
The interactions between the EF-hand domain of FimA (FimA-EF-N81Ter) and the H5 peptide, W7, and TFP are monitored by ^1^H, ^15^N-HSQC. The chemical shift or peak volume changes of well separated signals are fitted to obtain dissociation constant (Kd) of binding.

Next, we have investigated the interaction between FimA-EF-N81ter and a 14-residue synthetic peptide which encompasses the H5 region of FimA-EF (see Methods section) using NMR techniques (Fig. 6). By titrating ^15^N-labeled FimA-EF-N81ter with the H5 peptide, we observed substantial chemical shift changes in the HSQC spectrum of FimA-EF-N81ter in a fast exchange manner on the NMR time scale, indicating a relatively weak interaction. We could determine the dissociation constant value by fitting the chemical shift changes to a Kd of 3.0 × 10^–4^ M, which is much weaker than expected from the previous report for LPL (3.5 × 10^–6^ M)^34^. This can likely be attributed to the much shorter H5 of FimA compared to that of LPL. When the chemical shift changes are mapped on the model structure, all influenced residues locate in or around the hydrophobic pocket, supporting the overall model structure (Supplementary Fig. S4).

### Isothermal titration calorimetry (ITC) experiments

**Figure 7:**
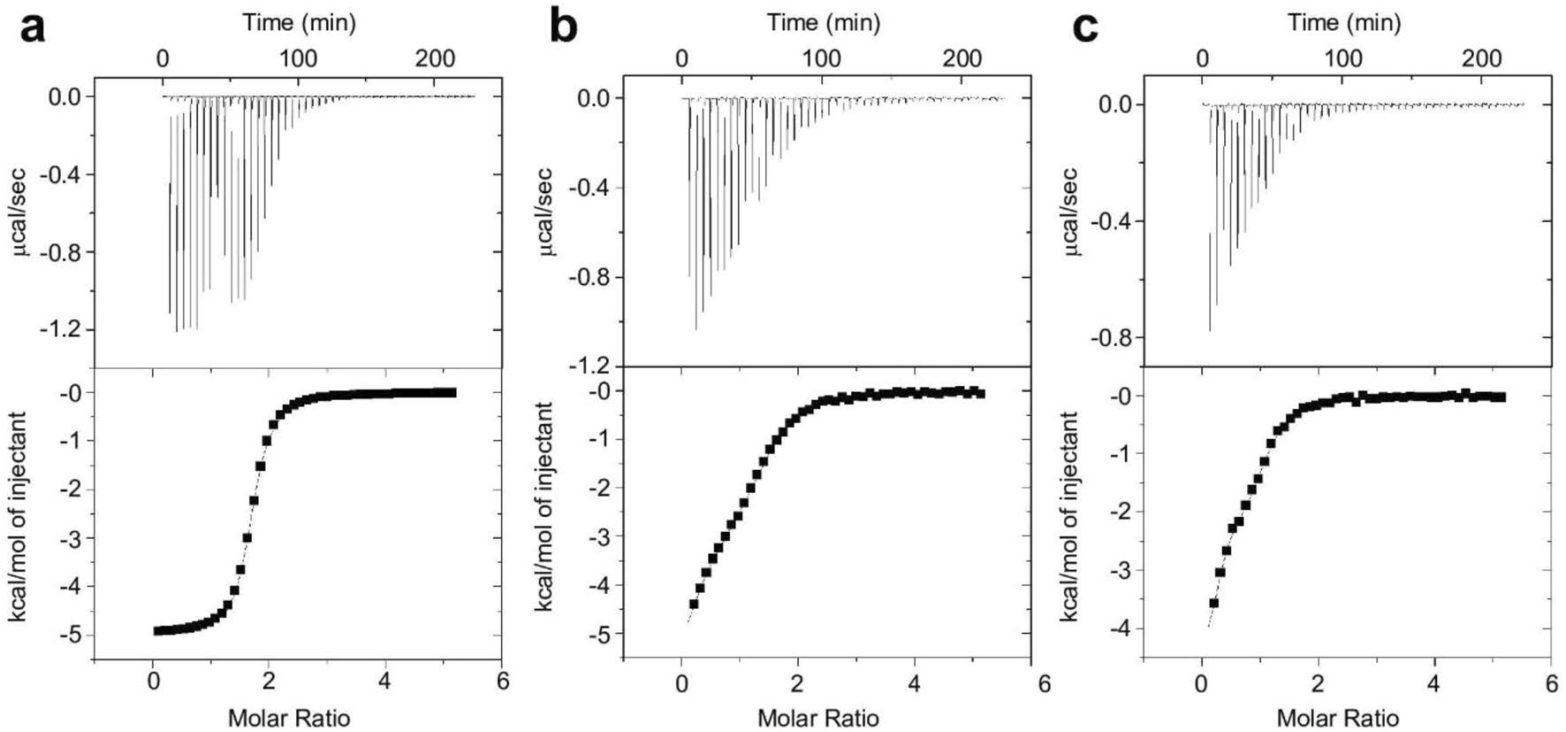
Calorimetric titration of FimA-EF (**a**), FimA-EF-N81Ter (**b**), and FimA-EF-F86A (**c**). The baseline corrected ITC titrations (top panel) and the fitted binding isotherms (bottom panel) are shown.

In order to study the calcium-binding properties of FimA, we used ITC to monitor the binding of calcium to the FimA constructs. Figure 7 shows the ITC isotherms obtained for the FimA-EF, FimA-EF-N81ter, and FimA-EF-F86A protein constructs. The ITC parameters derived from the curve fittings for all three constructs are summarized in Table 1. The isotherm obtained for the FimA-EF could be fit with one set of site model and produced a single Kd value of 0.6 µM and a stoichiometry of 1.7 (Fig. 7 and Table 1). Therefore, the binding of two calcium ions seem to be cooperative and the Kd value agrees with the previous observation that 200 µM calcium can almost fully turn off the actin-bundling activity of this protein^40^. On the other hand, for the other two constructs a two sets of sites model was required to fit the data. Two distinct calcium binding events are clearly visible in their binding isotherms. The Kd values obtained for FimA-EF-N81ter were 0.5 and 3.7 µM. These results resemble those obtained for human LPL^34^, and it suggests that the H5 region of FimA also modulates its calcium binding properties. It seems that the H5 is required to achieve the cooperativity between the two binding sites by enhancing the calcium binding affinity of the weaker site to the low uM range. From our current data, it is uncertain which EF-hand motif is the one that is modulated by H5 as both calcium binding loops possess proper amino-acid sequence for calcium binding (Fig. 1 and^35^). Nonetheless, our NMR data show that the first EF-hand motif (EF1) undergoes substantial structural changes upon H5 peptide binding (Supplementary Fig. S4). This suggests, but does not prove, that EF1 is the weaker site and requires the enhancement by the H5 binding to gain functional calcium affinity. We obtained essentially similar results for the FimA-EF-F86A construct with two distinct Kd values of 0.4 and 3.0 µM. This agrees with our ANS fluorescence results, showing that F86 is very important for the interaction between the H5 and the EF-hand domain.

**Table 1:** ITC derived thermodynamic parameters for the Ca^2+^-binding to FimA constructs.

Constructs	N1	K_d_1 (μM)	H1 (kcal/mol)	S1 (cal/mol/K)	N2	K_d_2 (μM)	H2 (cal/mol)	S2 (cal/mol/K)
WT	1.7^a^	0.6 ± 0.1^b^	–4932 ± 55	12.2 ± 0.6	–	–	–	–
F86A	0.3	0.4 ± 0.6	–4206 ± 50	15.5 ± 1.0	0.9	3.0 ± 1.0	–1788 ± 28	–19.3 ± 1.0
N81ter	0.3	0.5 ± 0.3	–6524 ± 71	7.3 ± 1.0	0.9	3.7 ± 1.0	–3107 ± 35	14.7 ± 0.4

^a^Small stoichiometry obtained for the first binding sites could be due to possible calcium contamination.

^b^Average values and SD values were obtained from three independent ITC runs (*n* = 3).

### Effect of calmodulin antagonists

**Figure 8:**
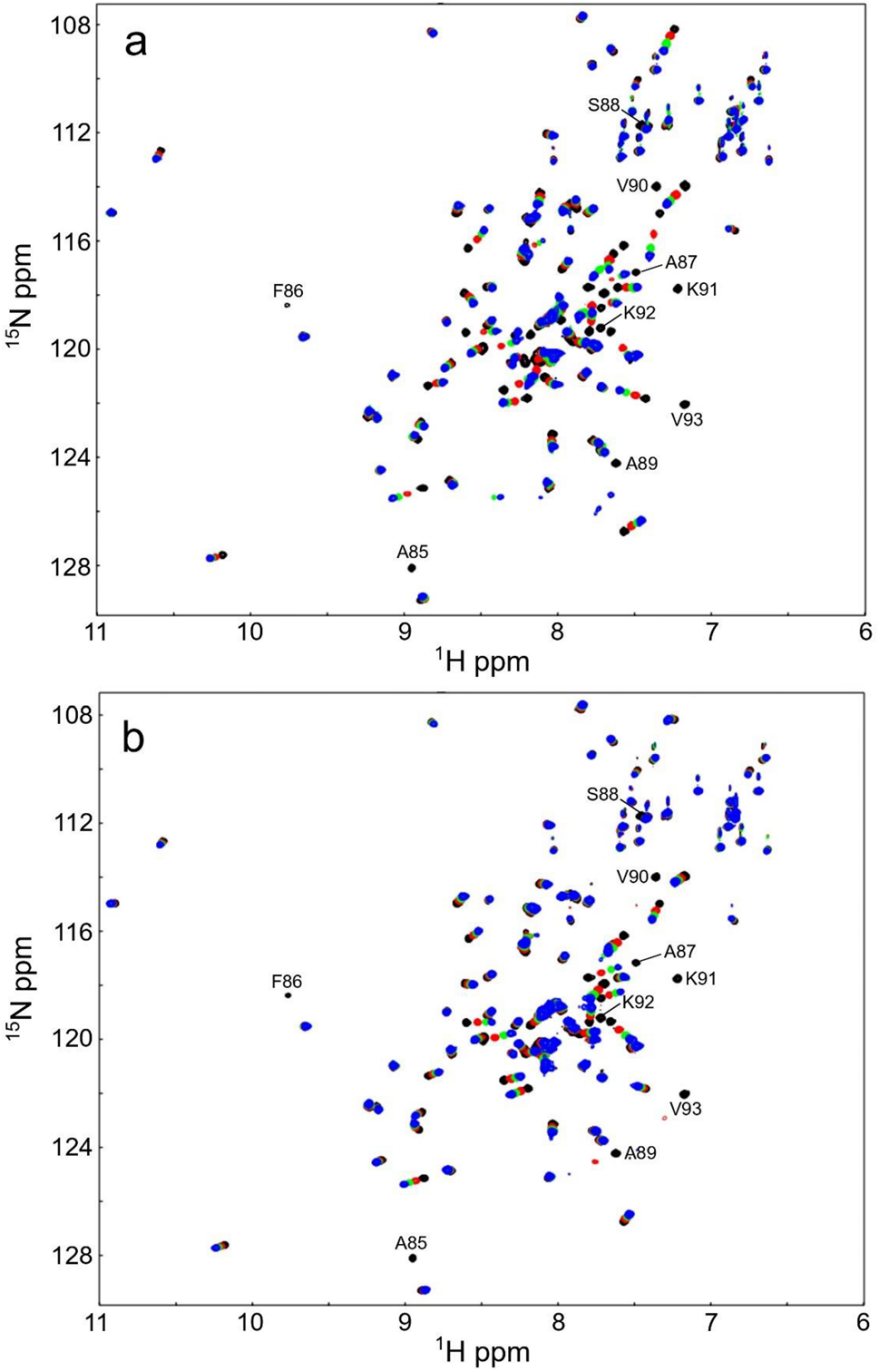
FimA-EF is titrated with TFP (top) or W7 (bottom). ^1^H, ^15^N-HSQC NMR spectra of FimA-EF with 0 (black), 0.5 (red), 1.0 (green), and 1.5 (blue) molar excess of TFP (**a**) or W7 (**b**) are overlaid. The signals from the H5 region are labeled.

Since the hydrophobic interaction between EF-hand domain and the H5 of FimA is rather weak, this interaction could potentially be disrupted by inhibitor drugs, which could lead to a deregulation of the Ca^2+^-sensitive actin-bundling of FimA. Therefore, we have tested the binding of two typical CaM antagonists, including W7 and TFP, to FimA-EF-N81ter. As expected from the similarity to the CaM domain, both drugs could bind to the FimA-EF-N81ter. The patterns of the chemical shift changes on the HSQC spectra upon binding to W7 and TFP are very similar to those observed with the H5 peptide (Fig. 6), indicating that these drugs bind to the same binding interface as the H5 peptide. However, W7 and TFP did bind with Kds of 3.8 × 10^–6^ M and 1.5 × 10^–6^ M, respectively (Fig. 6), which is two orders of magnitude stronger than the synthetic H5 peptide. This suggests that these drugs could possibly inhibit the interaction between the connected H5 region and the EF-hand domain of FimA. To confirm this, first, we titrated the H5 peptide into FimA-EF-N81ter in the presence of these drugs. No chemical shift changes were observed, indicating that the H5 peptide could not bind to the EF-hand domain as the binding interface is blocked by the drugs (Supplementary Fig. S5). Next, we titrated the FimA-EF construct with these drugs. Both TFP and W7 caused significant chemical shift changes in the HSQC spectrum of FimA-EF (Fig. 8). Particularly, the signals from the H5 region already disappeared when 0.5 molar equivalent of these drugs were added, indicating that the H5 region was dissociated from the EF-hand domain and no longer adopts a helical conformation. Thus, binding of W7 or TFP can displace the H5 region from the remainder of FimA-EF.

## Discussion

In this study, we have demonstrated that *Dictyostelium discoideum* FimA could function in a similar calcium regulatory manner as human LPL. Indeed, *Dictyostelium discoideum* has been used as a model organism to study dynamic actin filament organization as it is a highly motile organism and amenable to gene modifications^43^. Its genome contains 11 actin-bundling proteins^44^ and among them two are calcium regulatory proteins, namely FimA and a 34 kDa actin binding protein (ABP34). ABP34 also contains an N-terminal EF-hand domain which shows uM calcium affinity^45^ followed by three non-CH actin-binding domains^46,47^. Like FimA, Ca^2+^-binding to ABP34 negatively regulates the actin-bundling activity of the ABP34 protein^48–50^. Previously it has been shown that knockout mutation of one of these proteins did not alter the phenotype of the *Dictyostelium discoideum* amoeba, but that knocking out both proteins simultaneously resulted in abnormal phenotypes such as a small cell size and the formation of a small fruiting body^51^. Expressing a FimA construct that lacked the EF-hand domain did not rescue these phonotypes, suggesting that the Ca^2+^-regulated actin-bundling of FimA is important for these phenotypes. It seems that these two proteins (FimA and ABP34) have complementary roles in calcium regulated actin-bundling in the amoeba.

On the other hand, previous studies of the AtFim1, AtFim5 and yeast fimbrin proteins also indicate that these proteins do not respond to changes in the calcium levels in the micromolar range, and our NMR data confirmed that calcium binding does not occur and that calcium-dependent structural changes do not take place in these fimbrin EF-hand headpiece domains. Furthermore, the secondary structure predictions of these fimbrins as well as that of Tetrahymena fimbrin, which is also known to be insensitive to calcium^52^, do not find any evidence for the presence of an H5 region, suggesting that the presence of the H5, and the capacity to respond to changes in the calcium concentration go hand in hand. Interestingly, the headpiece domain is conserved in the vast majority of known fimbrins and plastins regardless of their functionality for Ca^2+^-binding^1^. Indeed, careful studies by Skau et al. indicate that plastins/fimbrins in yeasts and mammals play rather distinct roles^53^. Consequently, it seems that this region must play an important biological role. Be that as it may, it remains to be determined at a molecular level, what the exact role is for the non-calcium-binding headpiece regions that are found in several plastin and fimbrin proteins.

Our studies have demonstrated that *Dictyostelium* FimA can be regulated by calcium-binding to the EF-hand domain followed by the binding to the regulatory switch-helix (H5) in the same manner as the human LPL. Since *Dictyostelium*, leukocytes, and metastatic cancer cells all use amoeboid migration, this further supports the idea that the switch-helix is essential in a dynamic actin-bundling of fimbrins and plastins by responding to the calcium signalling that leads to the motility. LPL is known to be expressed ectopically during the transformation of a normal cell to a metastatic cancerous cell^54–59^ and it plays an important role in their motility. About 90% of cancer-related deaths is estimated to be caused by metastasis rather than by the original tumor. In recent years, various inhibitors towards fascin-1, another human actin-bundling protein that is also upregulated in metastatic cancerous cells have been synthesized (reviewed in^60^). On the other hand, to the best of our knowledge, inhibitors directly targeting LPL as a potential anti-metastatic drug have yet to be reported. We therefore suggest that this amoeba may become a valuable test organism for the development of novel drugs that aim to target the LPL involved in cancer metastasis.

## Methods

The synthetic gene constructs for the expression of the headpiece regions from fimbrins from *Arabidopsis thaliana* (AtFim1; residues 1–114, and AtFim5; residues 1–113), *Saccharomyces cerevisiae* (Sac6p; residues 1–99), *Schizosaccharomyces pombe* (Fim1; residues 1–102), and *Dictyostelium discoideum* (FimA; residues 1–93) were obtained from GeneArt (ThermoFisher) with codon optimization for expression in *Escherichia coli*. The headpiece regions of these fimbrins were determined according to sequence alignments with the headpiece domain of L-plastin (Fig. 1). All constructs were designed to include the two EF-hands as well as the potential regulatory switch-helix region. All the genes were subcloned in the inducible pET15b vector (Invitrogen) using NdeI/XhoI sites, which contains an N-terminal 6His-tag followed by a TEV protease cleavage site. We have also generated two FimA mutants including FimA-EF-N81ter in which the last 12 amino-acids (the putative regulatory domain) are deleted and FimA-EF-F86A using site-directed mutagenesis. A synthetic peptide (Ac-SANASPAFASAVKK-NH_2_) encompassing the sequence of the putative regulatory domain from FimA was obtained from Genscript; Its purity was over 95% as shown by mass spectrometry and reverse phase HPLC analysis. TEV protease was expressed and purified form the pRK793 plasmid (Addgene) as previously described^61^.

### Protein overexpression and purification

Overexpression of all the FimA constructs including FimA-EF, FimA-EF-N81Ter, and FimA-EF-F86A was achieved in BL21(DE3) *E. coli* cells, grown in 1 L of Luria Bertani (LB) medium with 100 μg/mL ampicillin at 37 °C. Once an optical density at 600 nm of approximately 0.6 was reached, cultures were induced with 1.0 mM Isopropyl β-D-1-thiogalactopyranoside (IPTG) for 4 h. For uniform isotope labelling of the constructs, expression was conducted in M9 minimal media, containing 0.5 g/L ^15^NH_4_Cl and 3 g/L ^13^C-glucose or ^12^C-glucose. Bacterial cells were harvested by centrifugation, and subsequently resuspended in buffer containing 20 mM Tris (pH 8), 100 mM NaCl, and 50 mM imidazole. The suspension was then homogenized and cells were lysed via French Press. Cell particulate matter was pelleted through centrifugation at 38,000×*g* for 45 min. The supernatant was then loaded onto an IMAC column (Cytiva), washed with the above resuspension buffer, and the purified protein was washed off with buffer containing 20 mM Tris (pH 8.0), 100 mM NaCl, and 300 mM imidazole. The protein is then dialyzed overnight in 4 L of buffer containing 20 mM Tris (pH 7.5), 100 mM NaCl, 1 mM β-Mercaptoethanol, and 0.5 mM EDTA. The N-terminal His-tag was cleaved using Tobacco Etch Virus (TEV) protease in the presence of 1 mM dithiothreitol (DTT) following dialysis for 1 h at 34 °C. Following cleavage, the mixture is loaded onto a cOmplete column (Roche) equilibrated with 20 mM Tris (pH 7.5) and 100 mM NaCl. The FimA-EF construct is collected in the flow-through, leaving the cleaved His-tag and TEV protease bound to the column. FimA-EF was prone to degradation during purification and necessitated further purification using a reverse-phase high performance liquid chromatography (RP-HPLC) with a Protein-R column (Nacalai Tesque Inc., Kyoto, Japan) in order to separate the intact protein from the degraded species. Purity was confirmed via SDS-PAGE gel (Supplementary Fig. S6). All the protein concentrations were estimated using the molar extinction coefficient of peptide bonds at A214 nm^62^.

### Nuclear magnetic resonance (NMR) spectroscopy

All NMR experiments were performed with a Bruker Avance 600 MHz NMR spectrometer operating at 25 °C. Main-chain NMR signal assignments for the apo and Ca^2+^-bound FimA-EF and FimA-EF-N81ter constructs were completed with two-dimensional ^1^H,^15^N-HSQC spectra as well as multidimensional HNCACB, CBCA(CO)NH, HN(CA)CO, HNCO, HNCA, and HN(CO)CA experiments. {^1^H}-^15^N heteronuclear NOE data were obtained using a 5 s train of 120° proton pulses. All NMR samples contained ~ 0.5 mM ^15^N or ^13^C,^15^N-labelled protein, 100 mM KCl, 20 mM Bis–Tris (pH 7.0), 10% D_2_O, 0.03% NaN_3_, 10 mM ^2^H-DTT, and 0.5 mM 2,2-dimethyl-2-silapentane-5-sulfonate (DSS) supplemented with either 1 mM EDTA or 5 mM CaCl_2_. The DSS was used as the reference peak to obtain the ^1^H, ^15^N and ^13^C chemical shifts. All NMR spectra were processed using NMRPipe/NMRDraw^63^, and analyzed using NMRView software^64^. The CSP values were calculated as a weighted average chemical shift difference for the^1^H and ^15^N resonances, using the equation CSP = √(D_HN_)^2^ + (D_N_/5)^2^. Chemical Shift Index (CSI) values were calculated using the Cα and C’ chemical shifts^65^. The program MOLMOL was used to generate molecular graphics^66^.

### Isothermal titration calorimetry (ITC)

ITC experiments were performed on a MicroCal VP-ITC microcalorimeter (Malvern) at 25 °C. 40 μM (or 100 μM) FimA in calcium-free 20 mM HEPES buffer pH 7.2, 100 mM KCl, and 1 mM TCEP HCl, was titrated with 1.2 mM CaCl_2_ solution in same buffer and salt components. Prior to each titration, the sample cell of the ITC was soaked in 5 mM EDTA solution, and was rinsed stringently afterwards with calcium free buffer which was prepared via 1 week incubation with Chelex chelating agent. Each protein sample was exchanged into calcium free buffer and passed through a Calcium Sponge S column (Life Technologies). Data was fitted using the one or two site model, as applicable, with the MicroCal Origin software to obtain dissociation constants (Kd), stoichiometry (n), and free enthalpy (ΔH) values.

### Fluorescence spectroscopy

Fluorescence spectra were acquired at room temperature on a Varian Cary Eclipse spectrofluorimeter. Fluorescence experiments with 8-Anilinonaphthalene-1-sulfonic acid (ANS) were conducted with samples containing 60 µM ANS with and without 60 µM FimA-EF, FimA-EF-N81Ter, or FimA-EF-F86A at pH 7.5 in the presence and absence of Ca^2+^. The excitation wavelength was 370 nm and the emissions were recorded from 400 to 600 nm using excitation and emission slit widths of 5 and 10 nm, respectively.

### Circular dichroism (CD) spectroscopy

CD spectra were collected on a Jasco J-810 spectropolarimeter (Jasco, Inc., Easton, Md.). All the samples contained 10 μM protein in 10 mM Tris buffer (pH 7.0) and 2 mM DTT. Far-UV CD spectra were collected between 260 and 185 nm in a 1 mm pathlength cuvette, using a 0.5 nm step size and a scanning speed of 100 nm/min. The bandwidth was set to 1 nm and the response time was set to 0.5 s. For each sample, 10 spectra were accumulated and averaged, then data smoothing in the Jasco Spectral Analysis tool was utilized to generate the final spectra.

### Supplementary Information

Supplementary Information.

## Data Availability

The datasets analyzed in this study are available from the corresponding author on reasonable request.
