# Supplementary material for: The Dictyostelium discoideum FimA protein, unlike yeast and plant fimbrins, is regulated by calcium similar to mammalian plastins
Source: Sci Rep. 2023 Sep 27;13:16208. doi: 10.1038/s41598-023-42682-1 (PMC10533516; doi:10.1038/s41598-023-42682-1)
Supplement: Supplementary file 1 — Supplementary Information. [file 41598_2023_42682_MOESM1_ESM.docx]

**The *Dictyostelium discoideum* FimA protein, unlike yeast and plant fimbrins, is regulated by calcium similar to the mammalian plastins**

**Hiroaki Ishida, Andrew G. Woodman, Naoya Kitada, Tomoyasu Aizawa, and Hans J. Vogel**

**Supplementary Figure S1**


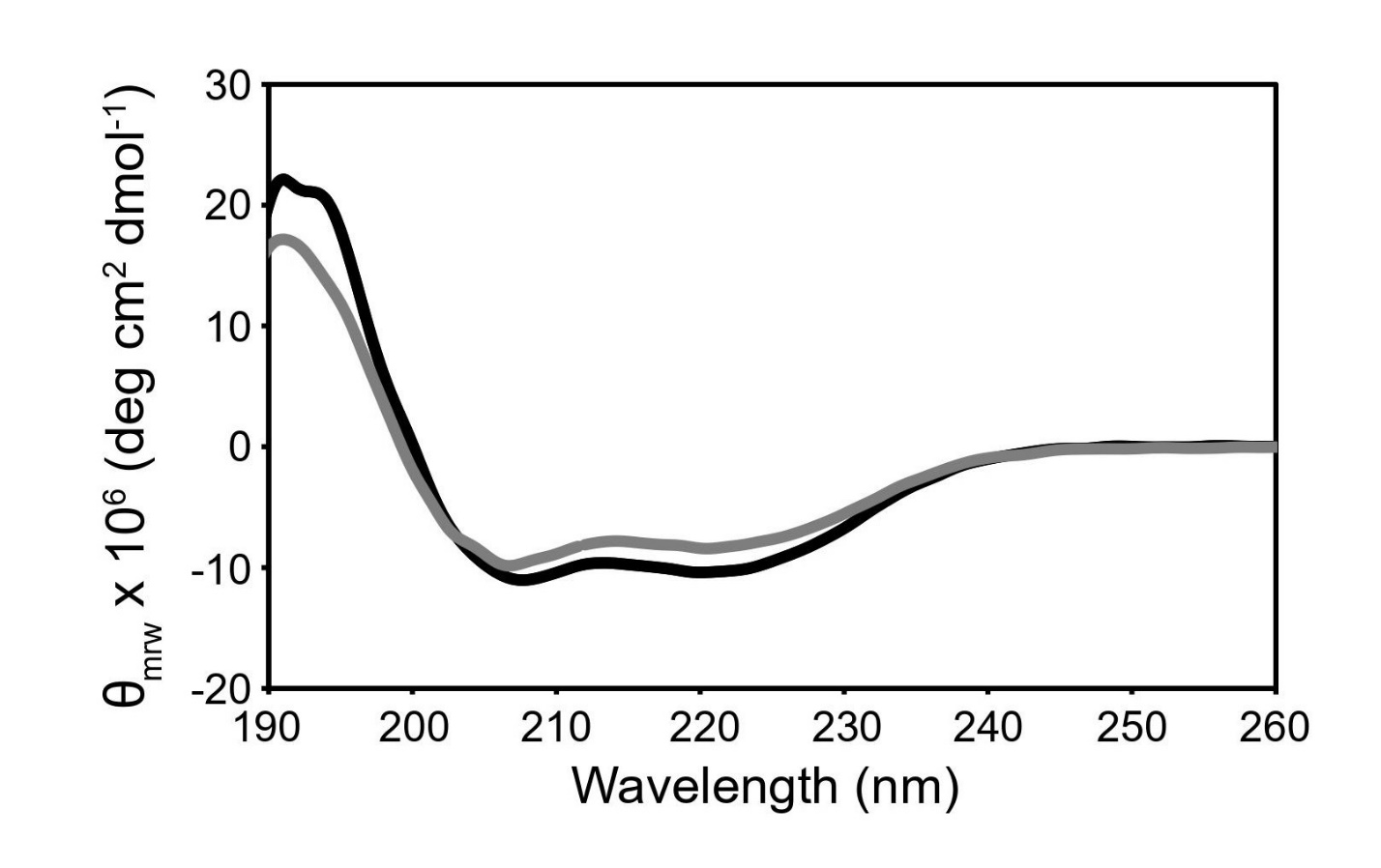


CD spectra of FimA-EF in the presence of EDTA (gray line) or CaCl_2_ (black line).

**Supplementary Figure S2**


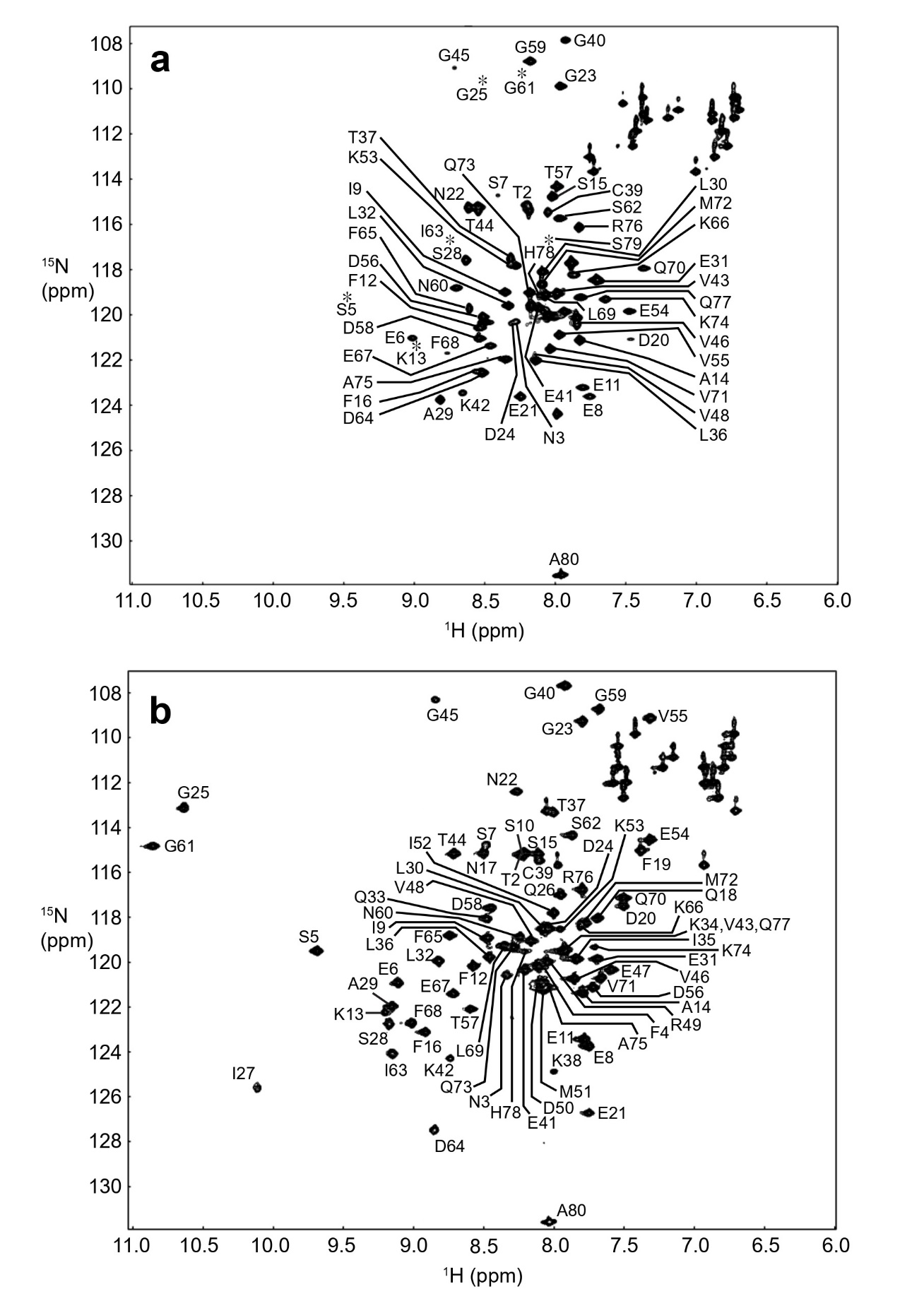


Assigned ^1^H, ^15^N-HSQC spectra of FimA-EF-N81ter in the presence of EDTA (a) and CaCl_2_ (b). The positions of signals that are invisible at this counter level are indicated by asterisks.

**Supplementary Figure S3**

**
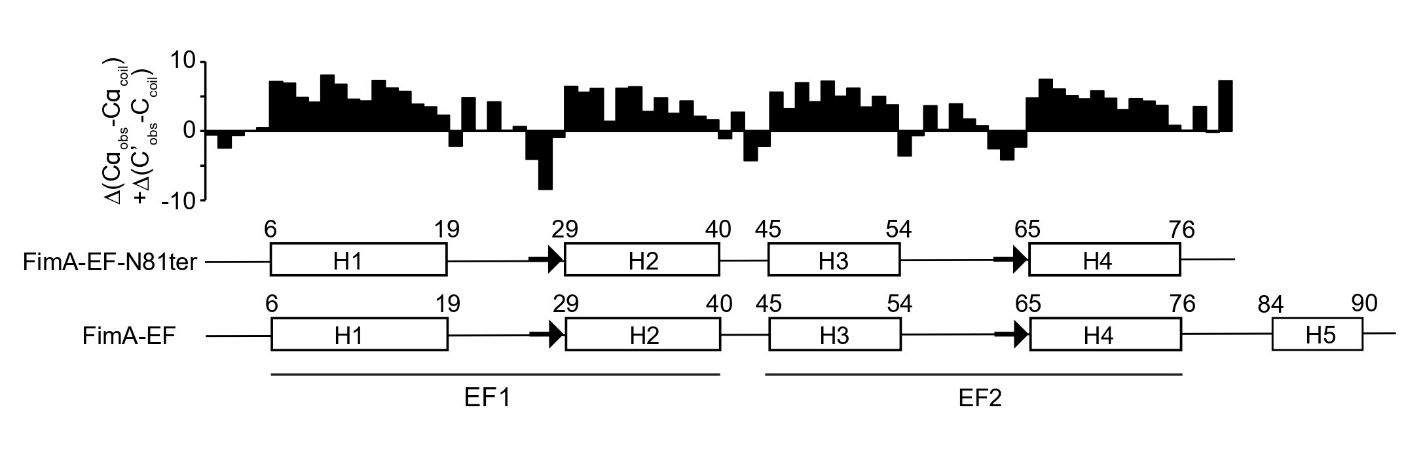
**

Chemical shift index (CSI) values for Ca^2+^-bound FimA-EF-N81ter are plotted as a function of the residue number (top). The secondary structures predicted from the CSI are also displayed and compared to those of FimA-EF (bottom).

**Supplementary Figure S4**


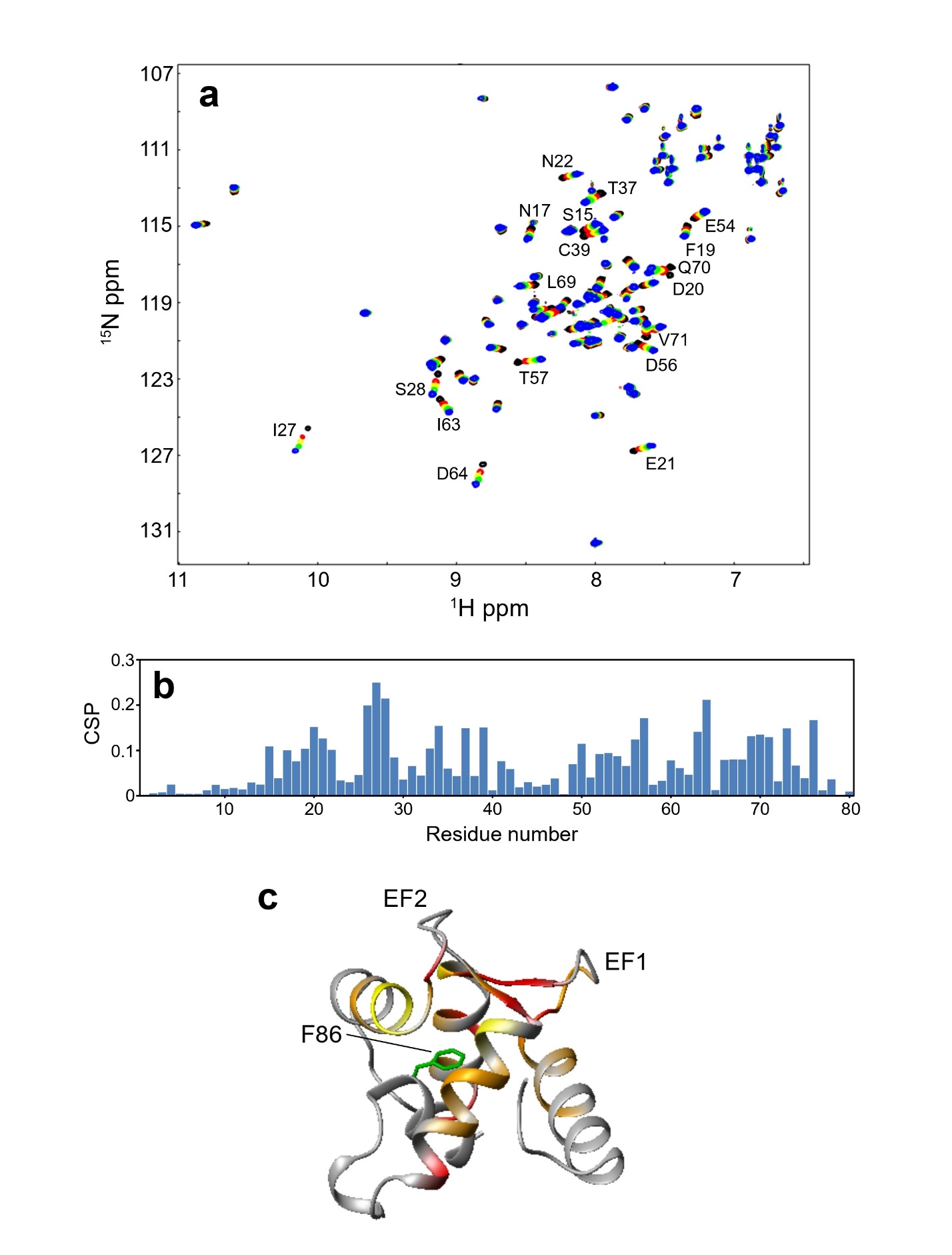


a, ^15^N-labeled FimA-EF-N81ter was titrated with 0 (black), 0.5 (red), 1.0 (yellow), 1.5 (green), and 2.0 (blue) molar equivalent of H5 peptide. The assignment for some peaks that show chemical shift changes are labeled. b, The chemical shift changes are plotted as the function of residue number. c, The regions with a large chemical shift change are highlighted in red (CSP ≥ 0.15), orange (0.15 > CSP ≥ 0.10), and yellow (0.10 > CSP ≥ 0.08) on the model structure of FimA-EF. The sidechain of the key hydrophobic F86 residue is also shown.

**Supplementary Figure S5**


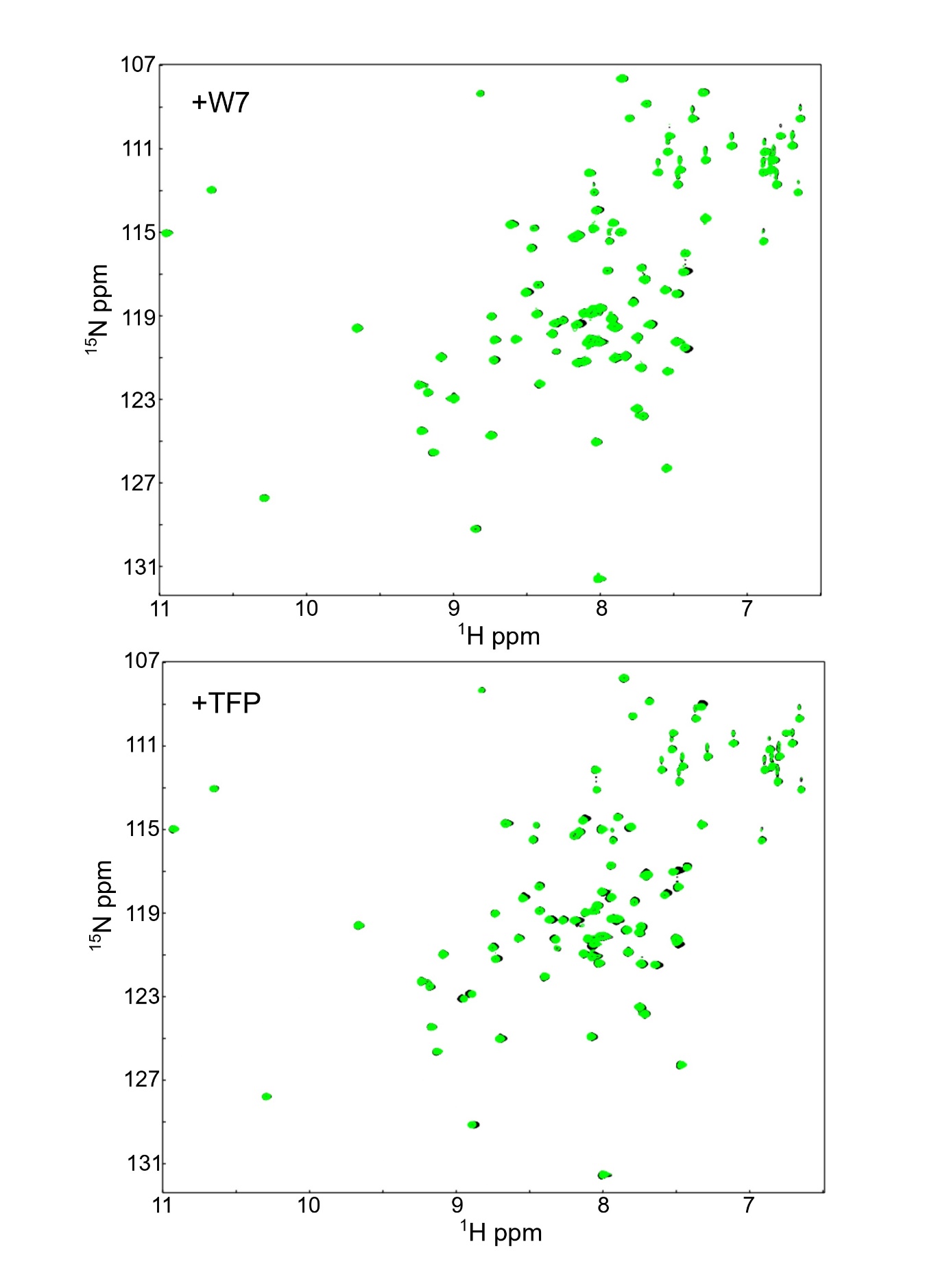


^15^N-labeled FimA-EF-N81ter was titrated with the H5 peptide in the presence of W7 or TFP. ^1^H, ^15^N-HSQC spectra of FimA-EF-N81ter with (green) and without 1.5 molar excess of H5 peptide (black) are overlaid.

**Supplementary Figure S6**


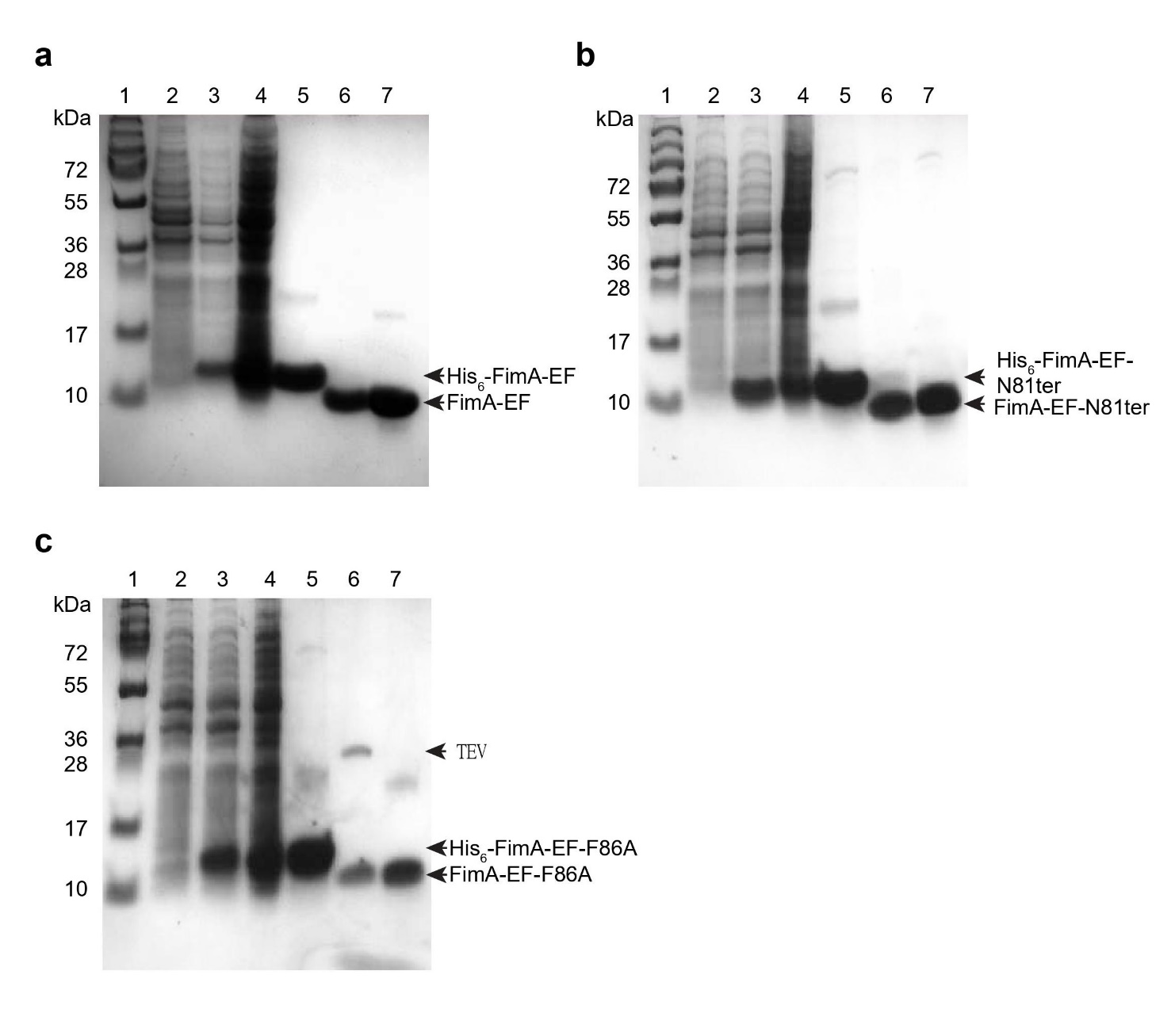
SDS-PAGE showing the expression and purification of (a) FimA-EF, (b) FimA-EF-N81ter, and (c) FimA-EF-F86A. In all panels, lane 1 shows the molecular mass marker. Lanes 2 and 3 represent the *E. coli* cell lysate before and after IPTG induction, respectively. Lane 4 represents the supernatant of *E. coli* lysate after centrifuge. Lane 5 represents the peak fraction eluted from the Ni^2+^-column. Lane 6 shows the protein after TEV digestion. Lane 7 shows the purified protein.
